# Longitudinal investigation of a xenograft tumor zebrafish model using polarization-sensitive optical coherence tomography

**DOI:** 10.1038/s41598-022-19483-z

**Published:** 2022-09-13

**Authors:** Antonia Lichtenegger, Junya Tamaoki, Roxane Licandro, Tomoko Mori, Pradipta Mukherjee, Lixuan Bian, Lisa Greutter, Shuichi Makita, Adelheid Wöhrer, Satoshi Matsusaka, Makoto Kobayashi, Bernhard Baumann, Yoshiaki Yasuno

**Affiliations:** 1 Computational Optics Group, University of Tsukuba, Tsukuba, Japan; 2 Center for Medical Physics and Biomedical Engineering, Medical University of Vienna, Vienna, Austria; 3 Department of Molecular and Developmental Biology, Faculty of Medicine, University of Tsukuba, Tsukuba, Japan; 4 Computational Imaging Research Lab, Department of Biomedical Imaging and Image-guided Therapy, Medical University of Vienna, Vienna, Austria; 5 Laboratory for Computational Neuroimaging, Athinoula A. Martinos Center for Biomedical Imaging, Massachusetts General Hospital and Harvard Medical School, Charlestown, MA USA; 6 Clinical Research and Regional Innovation, Faculty of Medicine, University of Tsukuba, Tsukuba, Japan; 7 Division of Neuropathology and Neurochemistry, Department of Neurology, Medical University of Vienna, Vienna, Austria

**Keywords:** Optical techniques, Imaging techniques, Biophotonics

## Abstract

Breast cancer is a leading cause of death in female patients worldwide. Further research is needed to get a deeper insight into the mechanisms involved in the development of this devastating disease and to find new therapy strategies. The zebrafish is an established animal model, especially in the field of oncology, which has shown to be a promising candidate for pre-clinical research and precision-based medicine. To investigate cancer growth in vivo in zebrafish, one approach is to explore xenograft tumor models. In this article, we present the investigation of a juvenile xenograft zebrafish model using a Jones matrix optical coherence tomography (JM-OCT) prototype. Immunosuppressed wild-type fish at 1-month post-fertilization were injected with human breast cancer cells and control animals with phosphate buffered saline in the tail musculature. In a longitudinal study, the scatter, polarization, and vasculature changes over time were investigated and quantified in control versus tumor injected animals. A significant decrease in birefringence and an increase in scattering signal was detected in tumor injected zebrafish in comparison to the control once. This work shows the potential of JM-OCT as a non-invasive, label-free, three-dimensional, high-resolution, and tissue-specific imaging tool in pre-clinical cancer research based on juvenile zebrafish models.

## Introduction

Breast cancer is a leading cause of death in female patients worldwide. In 2020, 2.3 million cases were diagnosed with breast cancer and 685,000 deaths occurred globally^1^. Modern therapy strategies enable to cure 70–80 % of patients if detected at an early stage. However, late stages and metastatic forms of breast cancer still evoke large challenges for current treatment options. Therefore, further research is needed to better understand the mechanisms involved in the development of breast cancer and its metastasis^2^.

Important approaches for the clinical treatment of breast cancer are precision or personalized medicine. They aim to improve the outcome of possible therapy options and at the same time try to reduce side effects for the patient^3,4^. Both of these, basic and clinical research fields rely on two emerging technologies. One being patient originated tumor models. They include in vitro cell cultures, such as conventional two-dimensional ones, spheroids, or organoids, and in vivo animal models from patient-derived tumor cells. The other research field is the detection of early biomarkers which enable a high-sensitive evaluation of these models. This allows to get deeper insights into the mechanisms involved in the disease and to test possible treatment options for new therapeutic targets^5^.

Among the models described above, in vitro cell cultures and spheroids allow for fast compound screenings, due to their small size and rapid creation time. However, they lack a physiological environment i.e., no tumor microenvironment, vasculature or active immune system is present^5^. In vivo animal models can be utilized to overcome this limitation.

The zebrafish is an established vertebrate model, in particular in the field of oncology and it has shown to be a good candidate for personalized medicine approaches^6^. This model organism has the required system complexity, and it is more time and cost-effective, due to its small size, fast maturation time and large offspring number, when compared to traditional rodent models^7^. Further, they share a high genetic and organ system similarity with humans and tumors formed histologically and genetically resemble patient ones^8,9^. In recent years genetic approaches have been well established in zebrafish, allowing to gain novel insights into the molecular genetics of various cancer types and advance in anti-cancer drug development processes^10^.

To investigate cancer growth in vivo in zebrafish, one approach beside the usage of transgenic models is to explore xenograft tumor models. Xenograft transplantation is the process of introducing living cells from one species into a host organism, such as the zebrafish, which allows to study a variety of human cancer cells without the need of establishing complex, new transgenic lines^11^. In zebrafish xenograft models, the tumor cells can either be injected into larvae or immunosuppressed juvenile or adult animals.

In most studies so far, larvae were utilized due to multiple reasons, such as that zebrafish lack an adaptive immune system at an early life stage, they are small and have a fast maturation time and are nearly transparent which allows for high-throughput compound screenings with conventional microscopic techniques^12^.

However, studies conducted in larvae are limited to a couple of days and for some tumor types and especially to observe metastatic processes a longer available experiment time would be beneficial. Furthermore, the water temperature in which larvae can optimally develop is around 28.5 $^{\circ }$C^11^. Literature has shown that by utilizing juvenile or adult fish, the water temperature can be increased to 37 $^{\circ }$C which facilitates human tumor cell growth. Finally, the administration and assessment of pharmacokinetics can be addressed in a more accurate way in juvenile or adult animals^11,13^.

In juvenile or adult zebrafish conventionally used microscopic techniques, such as white-light or fluorescence microscopy, have a limited penetration depth due to light scattering and absorption^14^. A semi-transparent zebrafish line was introduced to overcome this limitation, however leading to additional working steps and more complex breeding schemes^15^. To investigate non-transparent juvenile/adult xenograft zebrafish models a high-penetration, three-dimensional (3D) and high-resolution imaging technique is required.

Optical coherence tomography (OCT) is an optical imaging technique based on the interference of light, which enables the generation of 3D, high-resolution morphological representations of the investigated tissue based on the intrinsic contrast, i.e., the technique is label-free and non-invasive^16^. Additionally, OCT enables to investigate the vasculature by utilizing a technique called OCT angiography (OCTA)^17^. Polarization-sensitive OCT (PS-OCT) is a functional extension of conventional intensity-based OCT which additionally can analyze the polarization properties of a sample. Therefore, PS-OCT can add tissue-specific contrast in a label-free way^18^. Both conventional intensity-based OCT and PS-OCT have shown to be valuable tools in a wide range of microscopic applications^19–22^.

Conventional scatter intensity-based OCT setups have been used to investigate zebrafish at various development stages. Most of these studies so far were either focused on investigations in larvae^23–26^ or in adult zebrafish in the regions of the eye^27–29^ or the brain^30–32^. Yang et al. utilizing a polarization-sensitive OCT setup at a central wavelength of 840 nm to investigate various muscle groups and the skin structure of adult zebrafish^33,34^. Recently, our group showed first promising results in in vivo wild-type zebrafish at three development stages. We utilized our depth-resolved polarization-sensitive Jones-matrix OCT (JM-OCT) prototype operating in the infrared wavelength region to investigate zebrafish at 8-days, 1, and 2-months post-fertilization and characterized scatter and polarization properties of various organs^35^. In another study conducted in post-mortem adult transgenic tumor-bearing zebrafish, we showed the possibility of an improved tumor detection, compared to utilizing only the intensity-based information, using the same JM-OCT prototype^36^.

In this article, we present the investigation of an in vivo juvenile xenograft zebrafish model using the same JM-OCT prototype. Immunosuppressed wild-type animals at 1-month post-fertilization were injected with human breast cancer cells in the tumor group and with phosphate buffered saline (PBS) in the control group, respectively. Two studies were performed. In the first study, two control and four tumor-injected zebrafish (first generation) were investigated after 7 days post-injection with the JM-OCT prototype and the results were compared to the gold standard of histology. In the second longitudinal study, the number of animals was increased to 13 and 10 in the tumor and the control group (second generation) respectively and imaged in a non-invasive way over 21 days using the JM-OCT setup. The scatter and polarization changes in control and tumor-bearing animals were investigated and quantified. Furthermore, OCT angiography was used to examine the vasculature in these small animals. This work shows the potential of JM-OCT as a non-invasive, label-free, three-dimensional, high-resolution, and tissue-specific imaging tool in pre-clinical cancer research based on a juvenile zebrafish model.

## Results

The utilized polarization-sensitive JM-OCT prototype enables a multi-contrast examination of the anatomical features of the zebrafish. The three-dimensional tissue morphology was assessed by the intensity-based data. The depth-resolved birefringence was used as a marker for highly orientated structures such as the muscles. Further, to examine tissue samples introducing random changes in the polarization states, the degree of polarization uniformity (DOPU) was examined. This parameter has for example shown to be specific for highly pigmented tissue areas^18^. In addition, by using the OCTA information the vasculature was examined in a label-free way.

**Figure 1:**
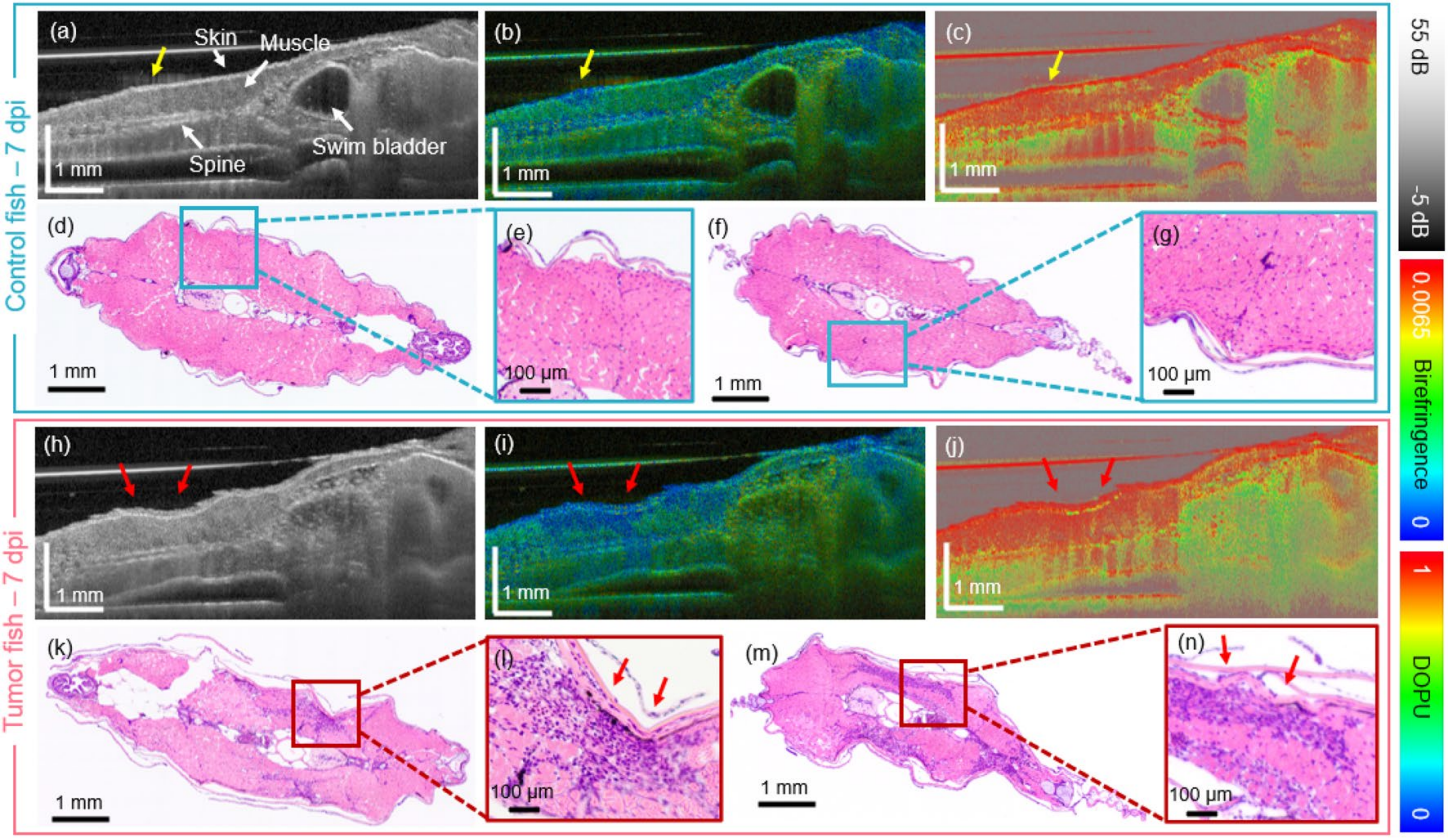
The OCT in comparison to histology results for the first generation of xenograft zebrafish at 7 days post-injection (dpi). (**a**–**c**) Scatter intensity, birefringence and DOPU sagittal cross-section images in a PBS injected control fish. The injection site is indicated by yellow arrows. (**d**–**g**) Corresponding transverse H &E-stained histology micrographs. (**h**–**j**) Scatter intensity, birefringence, and DOPU sagittal cross-section images in a tumor injected zebrafish. (**k**–**n**) Corresponding transverse H &E-stained histology images. The tumor location is indicated by red arrows.

**Figure 2:**
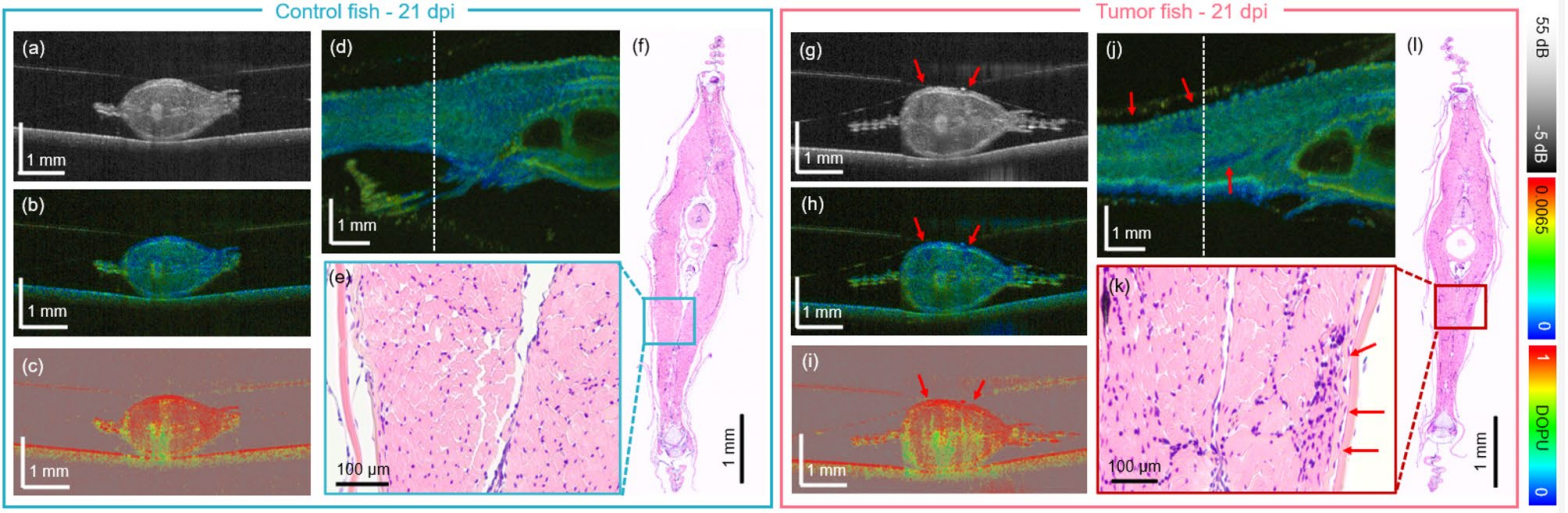
The JM-OCT in comparison to the histology results for the second generation of xenograft zebrafish at 21 days post-injection (dpi). (**a**–**d**) Scatter intensity, birefringence, DOPU transverse cross-sections and birefringence en-face image in a PBS injected control model. (**e**–**f**) H &E-stained histology micrographs in transverse direction and zoom-in image in the control animal. (**g**–**j**) Scatter intensity, birefringence, DOPU transverse cross-sections and birefringence en-face image in a tumor injected fish. (**k**–**l**) H &E-stained histology micrographs in transverse direction and zoom-in image in the tumor-bearing animal. The tumor location is indicated by red arrows. The locations of the transverse sections are indicated by white dashed lines in the en-face images.

The JM-OCT prototype was utilized to investigate a first generation of juvenile xenograft zebrafish (Study 1) and the results were validated by the gold standard of histology and immunohistochemistry (IHC) staining (see Supplementary Figure 3). Additionally, non-injected control animals were investigated and representative results are shown in the Supplementary material. Figure 1 shows the results obtained in a representative control (Fig. 1a–g) and tumor injected (Fig. 1h–n) fish at 7 days post-injection (dpi), respectively. Cross-sectional JM-OCT scatter intensity, birefringence, and degree of polarization uniformity (DOPU) images of the control (Fig. 1a–c) and the tumor (Fig. 1h—j) model are presented. The PBS injection site (indicated by yellow arrows) in the control fish can be identified as a region of low birefringence (blue) in comparison to the surrounding healthy musculature.

Also the tumor region (indicated by red arrows) exhibited decreased birefringence, and a disruption of the musculature morphology was observed, see Fig. 1h,i. The corresponding H &E-stained micrographs at two anatomical locations, revealed the abnormal accumulation of cells (dark purple regions in Fig. 1k–n) in comparison to the control muscle region (Fig. 1d–g). The muscle region showed high DOPU values with some small regions of increased depolarization in the fish skin (Fig. 1c,j), probably due to the pigmentation. Furthermore, low DOPU values were observed in the bottom region of most images, which arise from multiple scattering at deeper tissue regions^36^.

In the second zebrafish generation (Study 2), the animals were investigated longitudinally over 21 dpi. To be able to follow individual animals over time, they were kept separately in six-well plates. A comparison of the histology and JM-OCT results in representative control and tumor injected fish at 21 dpi, are shown in Fig. 2. Please note that after 21 days, both the control and tumor-injected animals showed a loss in muscle area (see Supplementary material). The scatter intensity, birefringence and DOPU-based results of a representative control fish are presented in Fig. 2a–d. A corresponding H &E-stained histology micrograph and a magnified image are shown in Fig. 2e–f. The scatter intensity, birefringence and DOPU-based results of a representative tumor-bearing fish are presented in Fig. 2g–j and the corresponding H &E-stained histology micrograph and magnified image are shown in Fig. 2k,l, respectively. The tumor locations are indicated by red arrows.

**Figure 3:**
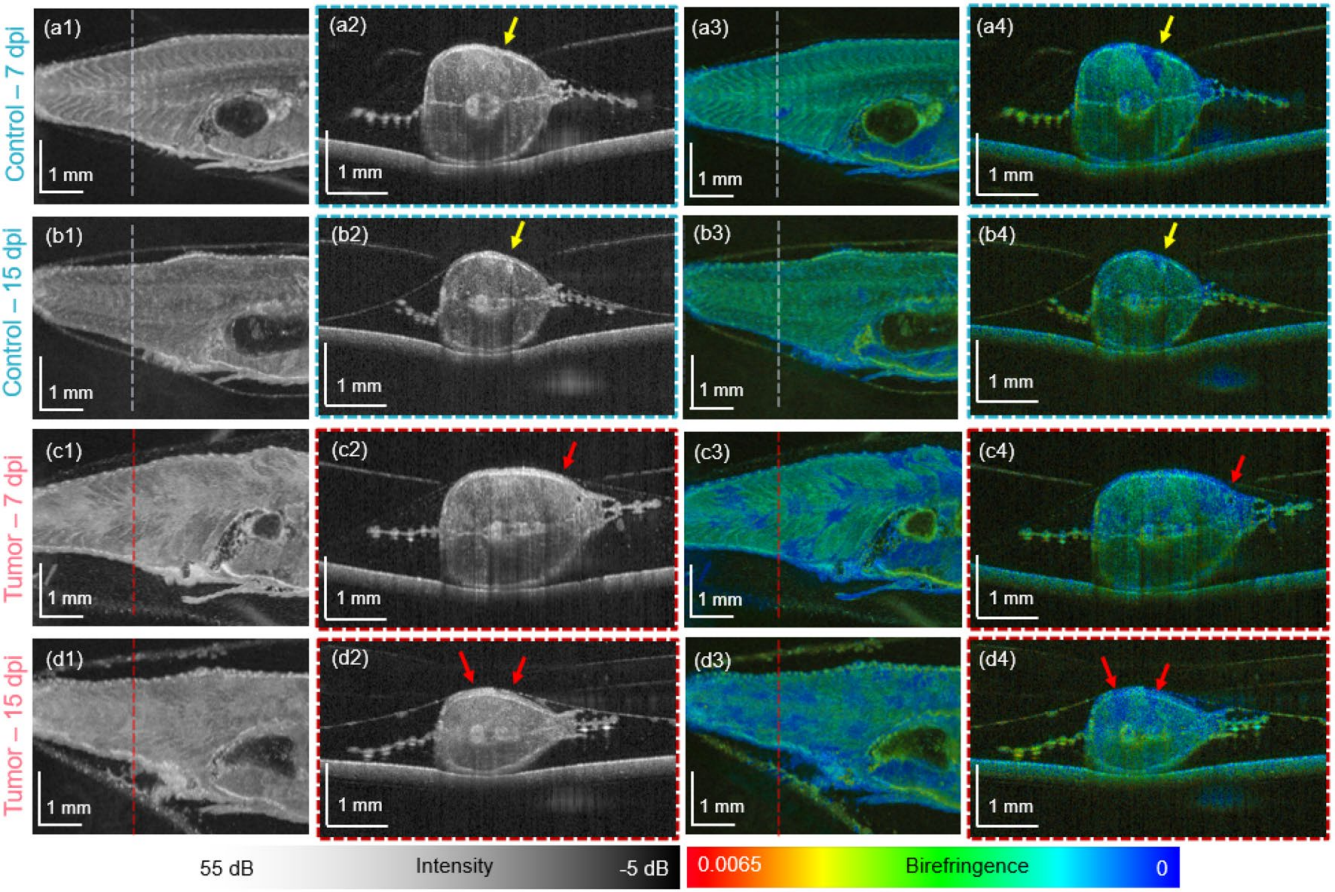
The scatter and polarization JM-OCT results over time in a control (**a1**–**b4**) and a tumor injected zebrafish (**c1**–**d4**). (**a1**–**a2**) Scatter-intensity en-face and B-scan images in a control fish at 7 days post injection (dpi). (**a3**–**a4**) Corresponding birefringence en-face and B-scan images. (**b1**–**b**2) Scatter-intensity en-face and B-scan images in a control fish at 15 dpi. (**b3**–**b4**) Corresponding birefringence en-face and B-scan images. (**c1**–**c2**) Scatter-intensity en-face and B-scan images in a tumor fish at 7 dpi. (**c3**–**c4**) Corresponding birefringence en-face and B-scan images. (**d1**–**d2**) Scatter-intensity en-face and B-scan images in a tumor fish at 15 dpi. (**d3**–**d4**) Corresponding birefringence en-face and B-scan images.

Figure 3 shows the scatter intensity and birefringence-based results of a control (Fig. 3a1–b4) and a tumor injected (Fig. 3c1–d4) animal at two time points (7 and 15 dpi, study 2), respectively. Additional results from 7 dpi until 21 dpi, obtained in a control and a tumor-injected animal are presented in the Supplementary Figure 4. In the control animal, the morphology of the musculature can be identified in the intensity-based data, see Fig. 3a1–a2 and further characterized by increased birefringence, see Fig. 3a3–a4. Remarkably, the PBS injection site in the control animal which was barley visible in scatter intensity data (Fig. 3a2), indicated by yellow arrows, shows a clearly decreased birefringence signature (Fig. 3b4). After 15-days the injection site is barely visible in the intensity-based data (Fig. 3b1–b2) and exhibited a small area of increased scattering and birefringence (Fig. 3b4), probably due to tissue scaring.

In the tumor injected zebrafish, after 7 days post-injection, an inhomogeneous scatter intensity pattern (Fig. 3c1–c2) and multiple regions of decreased birefringence (Fig. 3c3–c4) were observed. After 15 days post-injection the structural loss of the musculature in scatter intensity-based data (Fig. 3d1–d2) and larger areas of low birefringence (Fig. 3d3–d4) were revealed.

**Figure 4:**
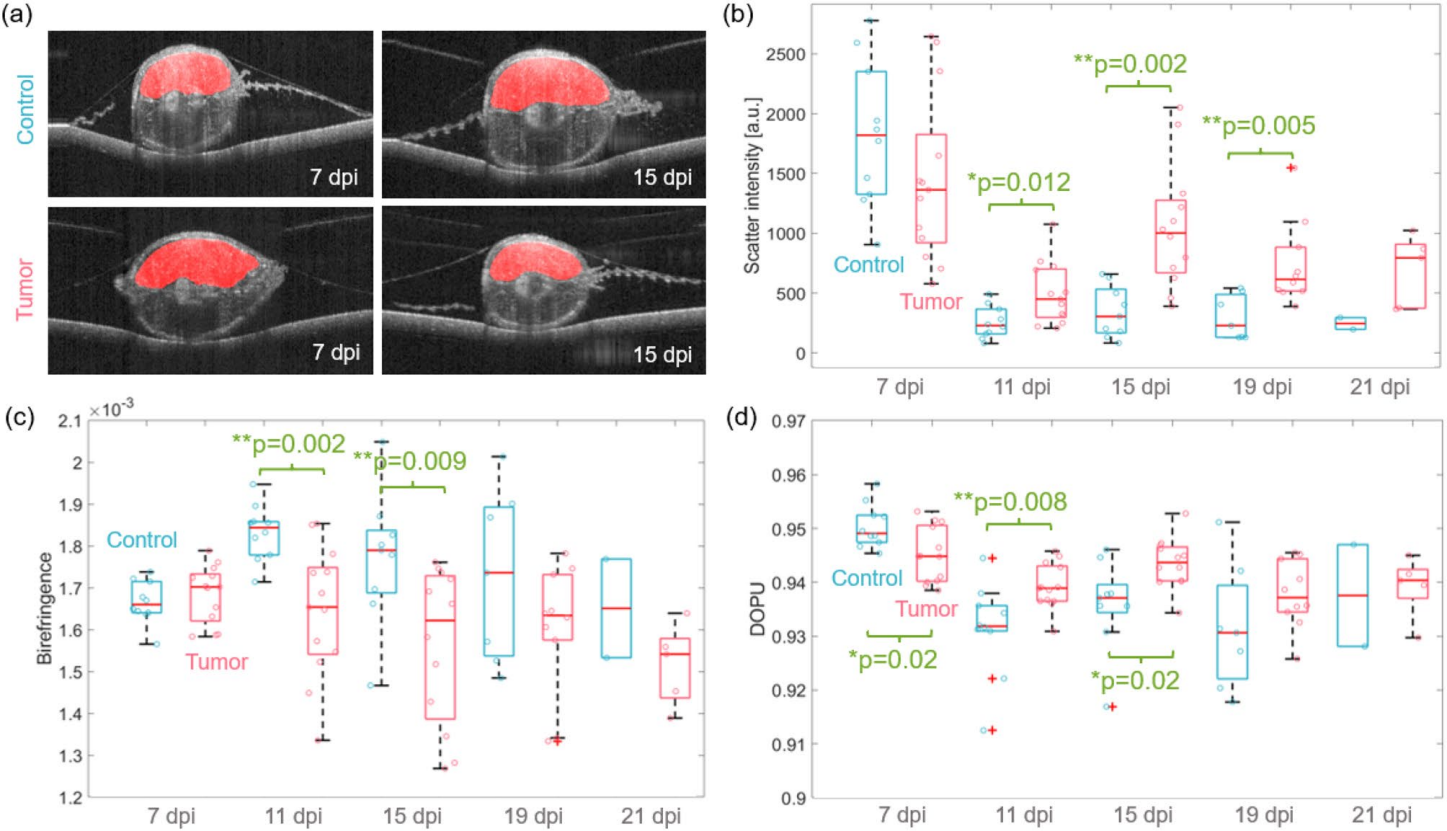
The scatter and polarization analysis. (**a**) Representative scatter-intensity B-scan images of control and tumor zebrafish overlaid with the automatic muscle segmentation results (red). (**b**) Box-whisker plots of the mean intensity values over the five measurement days post injection (dpi) in control and tumor animals. (**c**,**d**) Box-whisker plots of the mean birefringence and DOPU values. Each data point represents the mean value of the respective quantity for each animal analyzed.

Utilizing the Zebrafish Segmentation Net as described in the method section, the upper muscle regions were automatically segmented in the PBS (control) and tumor injected fish for the second xenograft generation (Study 2). Representative segmentation results in control and tumor models at day 7 and 15 are shown in Fig. 4a. Due to multiple scattering, reduced intensity and increased birefringence values were observed in the lower half of the fish tail musculature (Fig. 3c2,c4). That is why the evaluation of the scatter and polarization parameters was only performed for the upper muscle region. Utilizing the segmentation data and the scatter intensity, birefringence and DOPU values a quantitative analysis for all time points was performed. Figure 4b–d shows the box-whisker plots of the mean intensity, birefringence, and DOPU values over the five measurement dates in control and tumor animals. Single data points represent the mean values of each quantity for individual fish analyzed. Please note that the horizontal position of the points in a group was randomly selected for better visualization.

A general trend towards a higher scatter intensity, lower birefringence, and higher DOPU values were found when comparing tumor and control zebrafish. Rank sum tests were performed to identify statistically significant (p < 0.05) differences between the control and tumor fish at each time point. For the scatter intensity-based data, significantly higher values in the tumor injected animals were observed at 11 (p = 0.012), 15 (p = 0.002) and 19 (p = 0.005) dpi. Statistically significant lower birefringence in the tumor models was revealed at 11 (p = 0.002) and 15 (p = 0.009) dpi. Finally, statistically significant lower DOPU values were found in the tumor models at 7 (p = 0.02) dpi followed by significantly higher values at 11 (p = 0.008) and 15 (p = 0.02) dpi.

**Figure 5:**
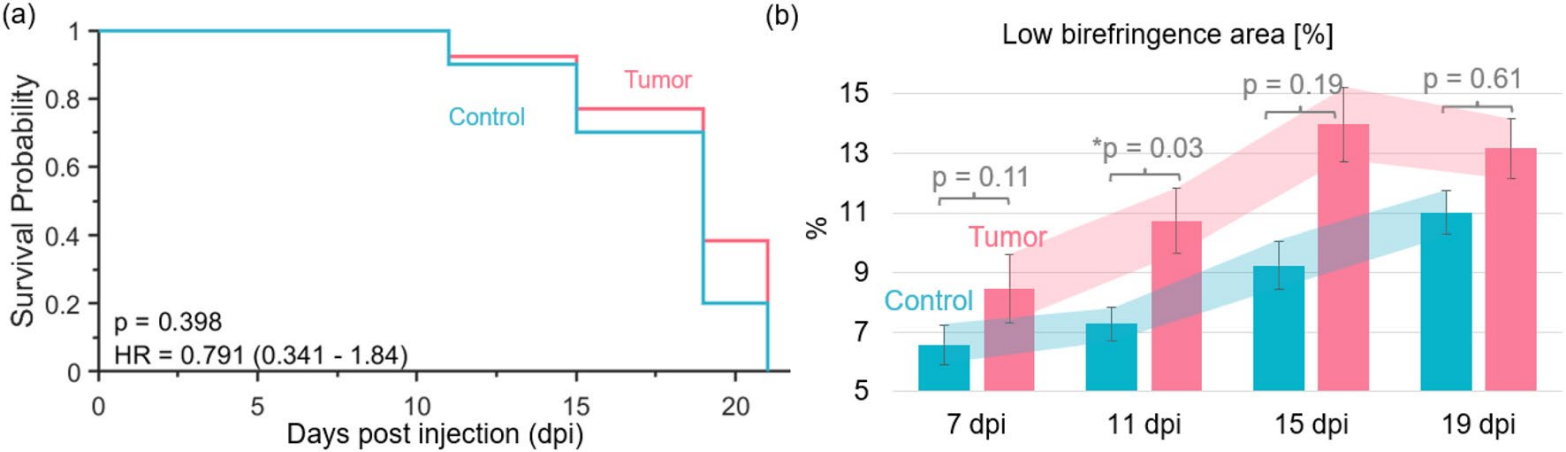
The survival and birefringence-based abnormality analysis. (**a**) Kaplan–Meier plot for the control and tumor zebrafish (HR—hazard ratio with 95% confidence intervals and the p-value of the log rank test are indicated). (**b**) Percentage of abnormal now birefringence values in the muscle region over the days post injection (dpi) with standard deviations indicated by vertical bars.

In the second study, 10 PBS-injected control and 13 tumor-injected animals were investigated in total. During the 21 days of the experiment around 90% of all animals survived until 15 dpi. However, in the last imaging session on day 21, only 20% (N = 2) of the control and 39% of the tumor-bearing fish were still alive. The respective survival Kaplan–Meier plots are shown in Fig. 5a. A hazard ratio of HR = 0.791 with a p value of 0.398 revealed no significant differences between the survival rate of control and tumor-injected zebrafish.

Further, the birefringence data acquired in the second longitudinal study were utilized to perform an abnormality analysis. By setting a threshold value of 0.0006 as abnormal low birefringence and by using the segmentation data, the area in percentage of these abnormal pixels in the muscle region was evaluated. The mean percentage values over 7–19 dpi are shown in the bar plot in Fig. 5b. The vertical bars indicate the corresponding standard deviations. The performed rank sum test showed a significant difference on day 11 (p = 0.03) and trends to larger areas of abnormal low birefringence on 7, 15, and 19 dpi.

**Figure 6:**
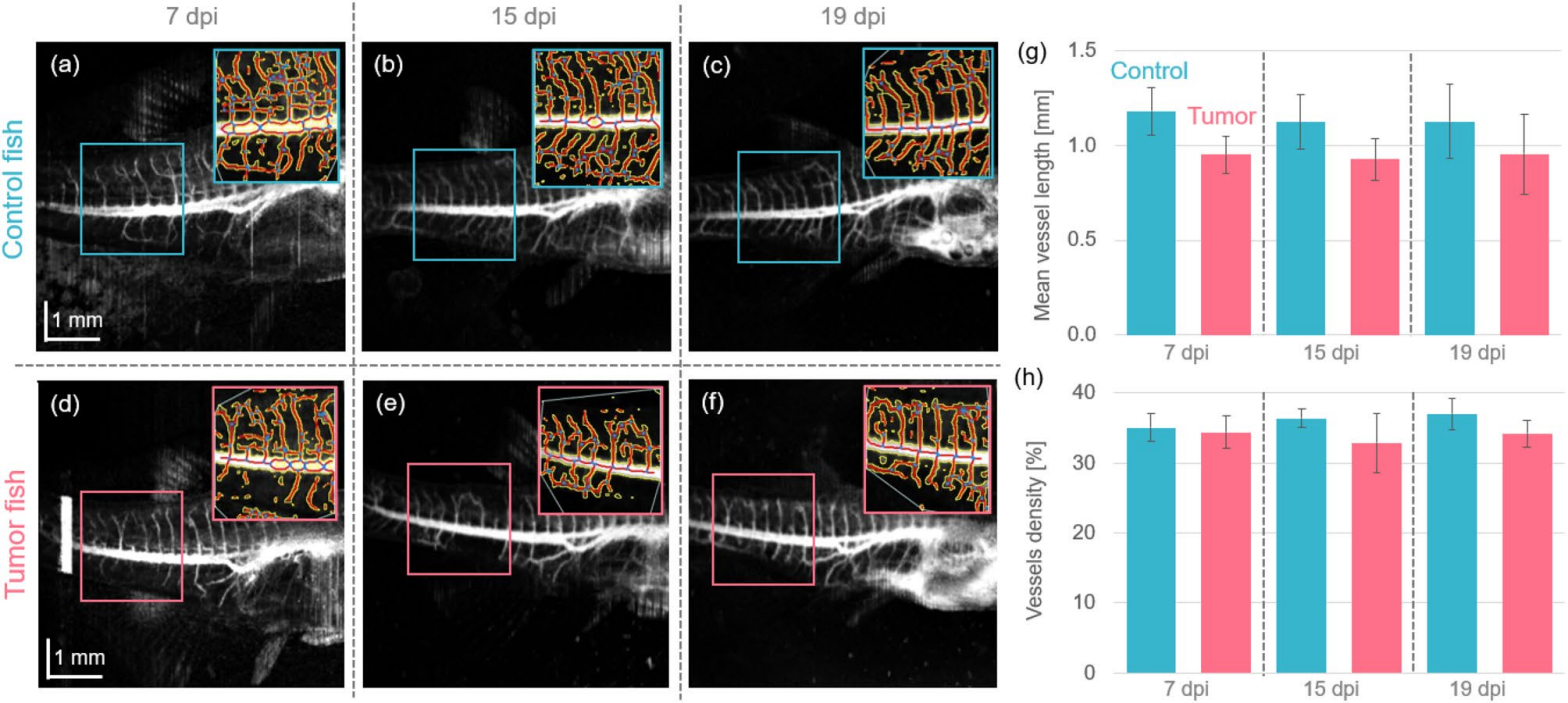
The OCTA data analysis. (**a**–**c**) OCTA en-face projections in control animals at 7-, 15- and 19-days post injection (dpi). (**d**–**f**) OCTA en-face projections in tumor injected animals at 7-, 15- and 19-dpi. The analyzed results obtained from AngioTool^37^ are included in the upper left corners. (**g**) The mean vessel length in control and tumor injected zebrafish. (**h**) The vessel density in percentage in control and tumor injected zebrafish.

Utilizing the OCTA data, the vasculature of the zebrafish can be investigated in a label-free way. Figure 6 shows representative en-face maximum OCTA projections and the vasculature segmentation results obtained with the AngioTool (inserted images in the upper right corners) of control Fig. 6a–c and tumor models Fig. 6d–f at day 7, 15 and 19 post-injection, respectively. When analyzing the mean vessel length and vessel density over the three time points, no statistically significant changes between control and tumor zebrafish were identified, see Fig. 6g,h, respectively. The tumor animals showed a trend of reduced vessel densities and mean vessel lengths at all time points, though these findings were not statistically significant.

## Discussion

A polarization-sensitive JM-OCT prototype operating at 1.31-$\upmu$m was utilized to investigate a juvenile zebrafish xenograft tumor model in a label-free and non-invasive way. In the tumor group, fish at the age of 1 month post-fertilization were injected with MCF-7 human breast cancer cells in the tail musculature. The control group was injected at the same location with a PBS solution. In the first study, the tumor-induced changes were evaluated at 7-day post-injection and the results obtained were compared to the gold standard histology. In the second longitudinal study, control and tumor fish were imaged every four until 21 days post-injection and at the end stage histology analysis was conducted. In comparison to conventionally used microscopic techniques, such as white light and fluorescence imaging, JM-OCT has the advantage that it is label-free, i.e., no exogenous contrast agent is needed and the tissue morphology over a couple of millimeters in depth can be investigated.

Study 1 revealed that an abnormal accumulation of cells in the tail musculature can be identified already after 7 days post-injection (Fig. 1k–n). We are planning to perform additional immunohistochemistry staining to further confirm these results. In the JM-OCT data a destruction of the muscle morphology with decreased birefringence values was found in comparison to the control zebrafish, see Fig. 1a,b and h,i, respectively. Literature has shown that healthy musculature in zebrafish exhibits increased birefringence values^33^. Further, a decrease in birefringence in diseased muscle tissue has been shown for murine and zebrafish models^36,38,39^.

Consistently, similar findings in scattering and polarization changes were observed in the second study, where the disruption of the muscle morphology and larger areas of lower birefringence values were observed on day 15 post-injection, see Fig. 3d1–d4 or 21 dpi, see Fig.2g–j.

To be able to follow changes in individual animals over time, each fish was kept in one chamber of a six-well plate. Keeping the fish over 21 days in this condition is stressful for the animals^40^, which was observed by a reduced movement and feeding behavior and it was also reflected in the JM-OCT and histology results, which may have led to muscle atrophy in control and tumor-bearing animals over time (Fig. 2 in comparison to Fig. 1), see Supplementary material. Also, the survival rate of the animals drastically dropped after 19 days post injection, see Fig. 5a, however no significant difference between the survival rate in the control and tumor group was observed. Long-term exposure to Tricaine anesthesia can lead to side effects such as stress, and it may be an additional factor for the observed reduced life expediency over time^41^. In a next step, we are planning to keep the zebrafish in standard one-liter tanks to improve the health condition during the longitudinal experiment. Furthermore, one limitation of the current experimental protocol was that no additional control zebrafish without any injection was investigated. This additional group will be included in the next study.

Utilizing the Zebrafish Segmentation Net the muscle regions in control and tumor zebrafish were automatically segmented. Utilizing these binary masks, the scatter and polarization property changes were quantitatively analyzed. A general trend towards higher scattering values in tumor versus control muscle areas was observed, with significant differences on 11, 15, and 19 dpi, see Fig. 4b. This increased scattering behavior might be caused by the more densely packed tumor mass in comparison to the low scattering muscle tissue^36^.

Lower birefringence at all time points, see Fig. 4c for the tumor-bearing in comparison to the control zebrafish was observed, with significant differences at 11 and 15 dpi. Furthermore, the birefringence data were utilized to compare the abnormal low regions observed in control and tumor animals. This analysis revealed that larger areas of abnormal low birefringence were found for the tumor models in comparison to the control animals at 7, 11, and 15 dpi with a significant difference at 11 dpi, see Fig. 5b. The decrease observed between 15 and 19 dpi, see Fig. 5b might indicate that in some cases the tumor mass could not properly manifest itself in the fish musculature. Furthermore, a trend towards an increase in these low birefringence values was also found in the control group, we believe this might be caused by muscle loss due to the abnormal moving behavior in the six-well plates.

Finally, the DOPU analysis showed significantly stronger depolarization (lower DOPU values) at 7 dpi, followed by lower depolarization (higher DOPU values) when comparing the control and tumor animals, see Fig. 4d. Wang et al. showed that human breast cancer exhibits high DOPU values in comparison to the surrounding fibrous-rich stroma tissue^42^. The authors stated that the lower DOPU values observed within the stroma, might be caused by the collagen bundles which are well-aligned in this tissue type resulting in significant changes in the polarization states. In contrary in the tumor area, the collagen content is very sparse, causing little-to-no change in the polarization state which leads to higher DOPU values. We believe that a similar effect can be observed in the tumor (higher DOPU values) compared to the muscle region (lower DOPU values) in our study. The same phenomena is also reflected in the birefringence data, where the disorganized tumor mass showed low values in comparison to increased once observed in the highly orientated muscle tissue, see Fig. 4c.

The scatter and polarization differences observed were most significant between 11 and 15 dpi, which might be caused by two main factors. First, the tumor mass needs some time to manifest itself in the fish musculature, which may be the cause for the clearer differences on 11 and 15 dpi. However, due to keeping the fish separately in the six-well plates, the feeding and moving behavior was abnormal, leading to a loss in body and muscle volume (see Supplementary material) which could lead to less significant differences at later time points. When analyzing the birefringence data in control animals (Fig. 4c), an increase followed by a slow decrease over time can be observed, which might be due to an inflammation response of the PBS injection and the worsened health condition over time. Please note that at 21 dpi, only 20% of control and 39% of tumor animals were still alive, that is why the statistical evaluation needs to be interpreted carefully. In the future we are also planning to investigate if there is a correlation between the measured optical parameters and the survival of the fish.

The quantitative analysis suggested that both scatter and polarization information are needed to fully characterize tumor-related changes in the juvenile xenograft zebrafish model over time. In a next step we are planning to explore further deep learning-based methods to analyze the generated contrast channels simultaneously for an improved identification of the tumor regions.

The OCTA analysis revealed no significant changes between the control and the tumor group in the vasculature over time, see Fig. 6. However, literature suggested that tumor growth is associated with angiogenesis, i.e., the abnormal growth of additional vessels due to the tumor mass^43^. We believe that multiple possible factors could explain why no changes were observed in this study. First, our setup was running at an A-scan rate of 50 kHz and an inter-scan time of 12.8 ms using 4 repetitions per B-scan location, resulting in an effective frame rate of 20 Hz to generate the OCTA contrast. The blood flow in vessels of zebrafish larvae was measured to be around 180 $\upmu$m/s with 160 bpm (beats per minute) or 2.67 Hz and adult animals exhibit slightly lower heart rates of around 110–130 bpm or around 2 Hz^44,45^. Additionally, anesthesia has shown to further decrease the heart rate^46^ and in general blood flow is irregular in tumor vessels, moving much slower and sometimes even oscillating^47^. This suggests that our setup might have a too short inter-scan time to be able to capture such slow motions. Second, the work of Stoletov et al. suggested, that the size of newly formed micro-vessels around the tumor are in the order of 10 $\upmu$m^43^. The axial and lateral resolutions of our JM-OCT prototype were 14.0 $\upmu$m and 18.1 $\upmu$m, respectively. This might not be sufficient to visualize the fine structures of these small developing vasculature^25^, and hence reduce the accuracy of the AngioTool analysis. Further a field-of-view of 6 $\times$ 8 mm was imaged by 512 $\times$ 512 pixels, resulting in lateral pixel resolutions of 11.7 $\upmu$m $\times$ 15.6 $\upmu$m, which might further degrade the accuracy of the analysis. Therefore, the optical resolution and sampling density needs to be increased in a follow-up study. We are planning to conduct a similar study using longer inter-scan times, an objective lens with a higher resolution capability, and a higher pixel density to overcome these limitations in the future. Last, one possibility is that the injected tumor cells did not yet form a new vasculature network and therefore no changes were detected in the OCTA data. It has been described in literature that especially at an early disease stage breast cancer can remain in a dormant state which does not stimulate angiogenesis^48^. In a next study, we are planning to validate our OCTA results by fluorescence imaging to gain a deeper understanding for the OCTA capabilities of the presented prototype.

So far, for both studies presented, the water temperature was kept at 28.5 $^{\circ }$C, which is the preferred habitat of zebrafish. However, literature suggested that keeping the zebrafish at 37 $^{\circ }$C, which is the optimum temperature for human cells, would facilitate the tumor growth^13^. That is why we are planning to evaluate the effect of increased water temperatures on the tumor growth and the animals in a next generation. Furthermore, the amount of tumor cells injected so far was based on previous literature^43^. In a next step, larger or even smaller amounts can be injected to explore the response differences.

To investigate tumor growth in juvenile/adult xenograft zebrafish, the immune system of the animals needs to be suppressed, that the host organism does not reject the introduced cells. Besides the usage of genetically modified animals, this can be done by radiation or chemical compounds such as Dexamethasone, as used in this study^11^. Mendonca-Gomes et al. recently showed that long-term treatment of Dexamethasone increases the engraftment efficiency of human breast cancer cells in adult zebrafish^49^. Though, at the same time the work of Ryu et al. showed that high concentrations of Dexamethasone can introduce muscle atrophy in zebrafish^50^. In the future, the influence of Dexamethasone on the tumor growth must be further investigated.

In the presented work, we explored a juvenile xenograft zebrafish model based on the injection of MCF-7 human breast cancer cells. In the future, we are also planning to include interventional groups to perform anti-cancer drug testing to further show the usability of our setup as a tool for precision-based medicine. The proposed JM-OCT prototype can further be used to investigate a wide range of other zebrafish tumor models, by exploring different cancer cell types and injection sites or even transgenic models. In conclusion, the presented work showed the potential of JM-OCT as a non-invasive, label-free, three-dimensional, and high-resolution tool for pre-clinical cancer research based on juvenile zebrafish models.

## Methods

### Tumor xenograft zebrafish model

**Figure 7:**
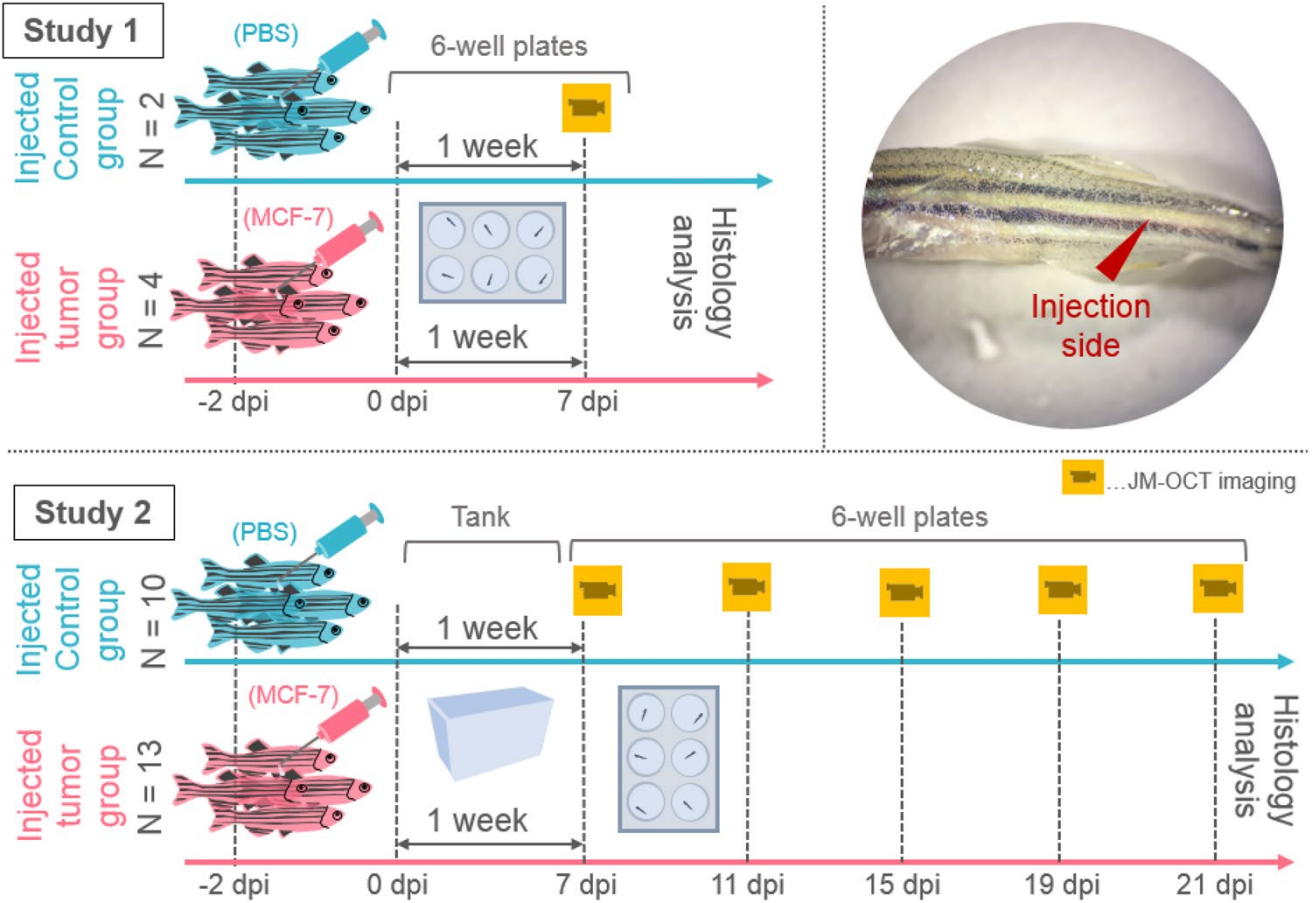
The two conducted xenograft zebrafish studies. In the first study, two control and four tumor-injected animals were investigated. In the second study, 10 and 13 control and tumor injected zebrafish, respectively were analyzed. A white-light photograph of the injection site in the tail musculature is shown (dpi—days post-injection).

Two generations of xenograft zebrafish were investigated, see Fig. 7. For both studies, AB (wild-type) zebrafish at the age of 1-month-post-fertilization were investigated. Zebrafish were grown under standard conditions with a 14-h day and 10-h night cycle^51^. Additionally, the water, which was kept at 28.5 $^{\circ }$C, was changed every day. The animals were anaesthetized using Tricaine (3-Aminobenzoic Acid Ethyl Ester Methane-sulfonate, 0.16 mg/ml) and were placed under a white-light stereo microscope. Using a pressure microinjector and a glass needle, phosphate buffered saline (PBS) in control and MCF-7 human breast adenocarcinoma cells diluted in PBS in tumor fish were injected into the tail musculature.

All animal experiments were performed in accordance with the animal study guidelines of the University of Tsukuba. All experimental protocols involving zebrafish were approved by the Institutional Animal Care and Use Committee (IACUC) of University of Tsukuba. The present study was designed, performed, and reported according to the principles of ARRIVE (Animal Research: Reporting of In vivo Experiments) guidelines.

#### First generation of xenograft zebrafish

For the first study (Fig. 7 Study 1) the zebrafish were immersed in Dexamethasone (FUJIFILM Wako Pure Chemical Corporation, Japan, 10 $\upmu$g/ml) for 2 days, a drug to suppress the immune system^49^. All animals were kept separately in six-well plates. Four zebrafish were injected with MCF-7 cells (concentration: $2 \times 10^6$ cells in 1 ml PBS) and in two control animals only the PBS solution was injected at the same location, see Fig. 7. After 7 days post-injection (dpi) all fish were imaged under anesthesia (Tricaine (0.16 mg/ml)) using the JM-OCT prototype. The fish were placed in a petri dish and each imaging session took less than 5 min, to ensure the survival of the animals. All zebrafish were sacrificed after OCT imaging with an overdose of Tricaine and fixated in 4% paraformaldehyde (FUJIFILM Wako Pure Chemical Corporation, Japan) for histological workup.

#### Second generation of xenograft zebrafish

For the second study (Fig. 7 Study 2) the zebrafish were again raised until 1-month-of-age and were kept in Dexamethasone (10 $\upmu$g/ml) for 2 days in two separated one-liter tanks (control and tumor fish). In total 13 tumor (MCF-7 human breast cancer cells in PBS: $5 \times 10^6$ cells/ml) and 10 injected control (PBS solution) fish were investigated.

Starting from 1 week after the injection, the animals were imaged under anesthesia (Tricaine (0.16 mg/ml)) using the JM-OCT prototype every 4 days until 21 dpi and were kept in six-well plates to be able to differentiate them throughout the experiment. After the longitudinal investigation all animals were sacrificed with an overdose of Tricaine and fixated in 4% paraformaldehyde for histological workup. Please note that some fish died at intermediate time points, these animals were also fixated in 4% paraformaldehyde for histological workup.

### Histology analysis

**Figure 8:**
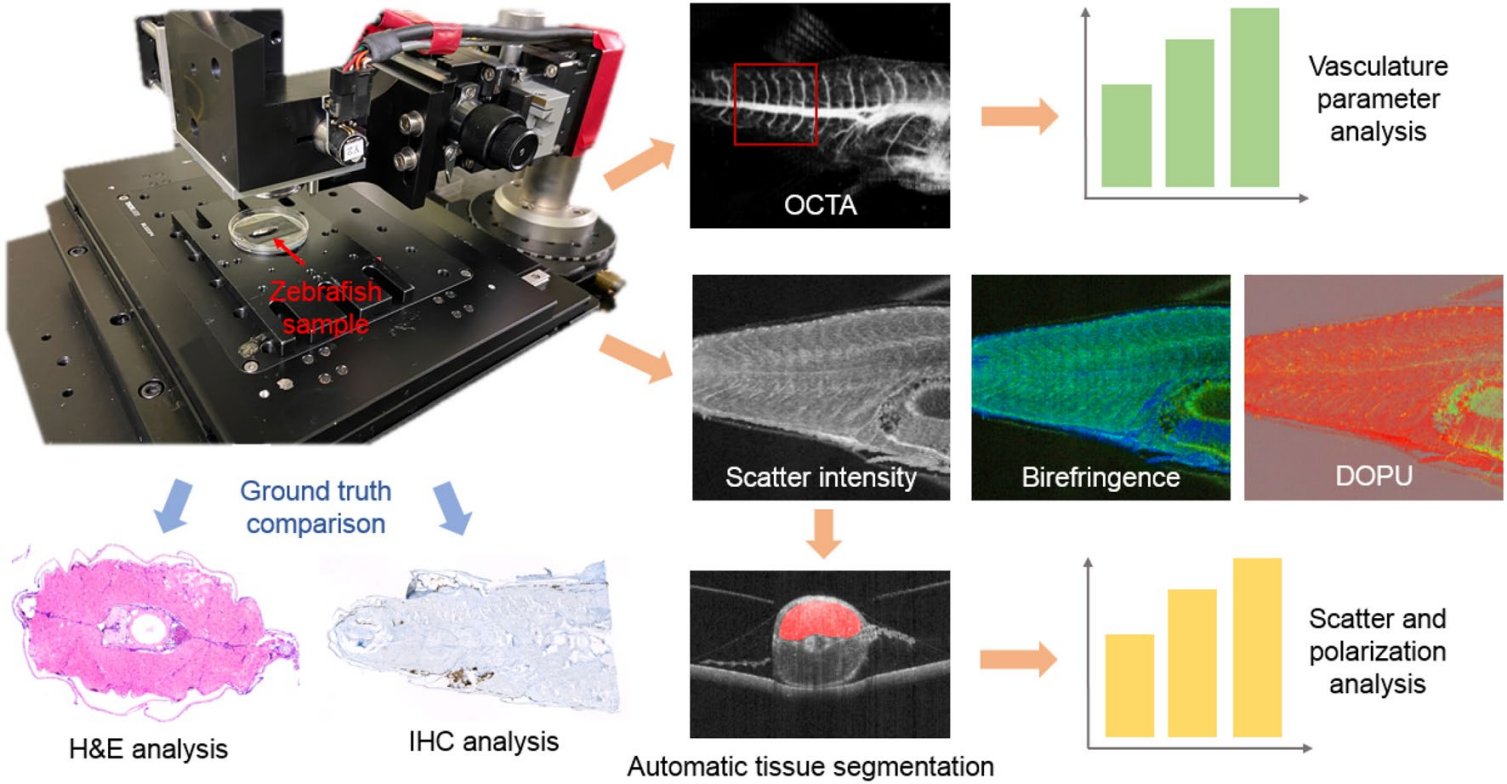
The data acquisition and processing steps with a photograph of the Jones-matrix OCT prototype. The scatter and polarization (birefringence and degree of polarization uniformity (DOPU)), and angiography (OCTA) data were analyzed. Quantitative analysis was performed based on an automatic tissue segmentation. As a ground truth histology analysis based on hematoxylin and eosin (H &E) and immunohistochemistry (IHC) staining was performed.

Following OCT measurements, histological workup was performed, see Fig. 8. Hematoxylin and eosin (H&E) stained micrographs were acquired with a standard transmission microscope (Olympus, SZX16) using 4$\times$, 10$\times$, and 20$\times$ magnification objective lenses. In addition, to confirm the presents of the MCF-7 cells in the zebrafish tail musculature immunohistochemistry was performed with an anti-HER-2/neu Rabbit Monoclonal Primary Antibody (6 $\upmu$g/ml, Roche). Diaminobenzidine was used as detection system.

### Jones-matrix OCT prototype

To investigate the in vivo zebrafish, a custom-built JM-OCT setup (Fig. 8) was utilized. Details of the setup can be found elsewhere^52^. A swept-source laser with a central wavelength of 1.31-$\upmu$m (AXA10823-8, Axsun Technologies, MA) was used for imaging and the JM-OCT prototype was based on a passive-polarization-delay-based PS-OCT scheme.

Images were acquired with an A-scan rate of 50 kHz and the system sensitivity was 104 dB with a probe beam power of 11 mW. The axial resolution in tissue was 14 $\upmu$m with a depth pixel separation of 7.24 $\upmu$m. The depth range in air was 2.9 mm. For imaging a scanning lens (LSM03 scanning lens, Thorlabs) featuring a focal length of 36 mm was used. Together with a beam diameter of 3.5 mm, the effective numerical aperture was 0.048, and it resulted in a lateral resolution of 18.1 $\upmu$m. Data sets comprised 512 $\times$ 128 or 512 $\times$ 512 lateral pixels with four repetitions per location for OCT angiography (OCTA) evaluation. The imaged field-of-view (FoV) was set to 6 mm $\times$ 8 mm at the injection site in the fish tail.

### JM-OCT data processing

Four contrasts were computed from the raw JM-OCT signal, which included the OCT scattering intensity, the local birefringence, the degree of polarization uniformity (DOPU) and the OCT angiography (OCTA) see Fig. 8. The image processing software Fiji^53^ was utilized for data visualization.

To generate the scatter intensity-based data (in the manuscript also referred to as the intensity data), the absolute-squared intensities of the four Jones matrix entries, corresponding to the four polarization channels, were averaged.

Different polarization states experience different speeds of light in a birefringent material. This effect can for example be observed in fibrous tissue structures such as muscles^18^. The depth-resolved birefringence data, also known as the local retardation, were obtained by a local Jones matrix analysis in combination with a maximum a posteriori birefringence estimator^54,55^. Please note that for the presented pseudo-color birefringence tomograms the scattering intensity, the birefringence, and the reliability of the birefringence estimation were combined. Therefore, the birefringence is expressed by color hue if it is reliable, otherwise the pixel is a shade of gray.

The random change of the incident polarization state at spatially adjacent sample locations is called depolarization or also polarization scrambling^56^. The randomization of polarization states is examined using the so-called degree of polarization uniformity (DOPU) and for the visualization a composite image of the scatter intensity and DOPU data was generated, leading to mostly grey pixel values in the background region^57^.

In vivo measurements enable the investigation of dynamic processes like the blood flow utilizing OCTA^17^. To analyze vasculature changes over time, three control and three tumor injected volumes at day 7, 15, and 19 dpi were utilized. To create the OCTA contrast, four repeated B-scans on one location were performed and a complex correlation-based approach was utilized^52^. First, the OCTA data were masked with the OCT intensity images to separate the background and valid tissue region. A three-dimensional Gaussian filter (standard deviation value of the distribution: 1.1, in all directions) was applied to the OCTA volumes. Maximum intensity projections over 30 pixels in depth (corresponding to 0.2 mm) in the muscle region at the injection site (200 $\times$ 150 pixels corresponding to 2.3 mm $\times$ 2.3 mm) were created. Using AngioTool (version 0.6a)^37^, an open-source software to analyze angiographic images, the mean vessel length and vessel density in the generated projection images were analyzed, see Fig. 8.

### Data evaluation

#### Survival analysis

To analyze the survival differences in the control and the tumor group over time a Kaplan–Meier plot was created. Further, the hazard ratio (HR) was computed based on Eq. (1).

1 $$\begin{aligned} HR=\frac{\sum O_C /\sum E_C}{\sum O_T/\sum E_T} \end{aligned}$$

O and E indicate the observed and the expected survived fish, respectively and C the control and T the tumor group. The hazard ratio is a measure of the magnitude of the difference between the two survival curves in the Kaplan–Meier plot, where values close to 1 indicate no differences between the two groups. Further, a log rank test, using a significance level of p< 0.05, was performed to evaluate if the observed difference between the groups was significant^58^.

#### Scatter and polarization evaluation

A neural network (Zebrafish Segmentation Net) was utilized to automatically segment the upper half of the zebrafish musculature of all control and tumor injected fish of the second study (in total 91 volumes each containing 128 images from five JM-OCT measurement sessions (7, 11, 15, 19 and 21 dpi)), see Fig. 8.

The Zebrafish Segmentation Net processes the volumes B-scan wise, i.e. the input of the network is an intensity-based transverse section and the output is a corresponding label map with float values. This map is then thresholded (values> 0.5 are set to 1) to obtain a binary label map. The architecture of the Zebrafish Segmentation Net is based on a U-Net architecture previously proposed by Ronnenberger et al.^59^, consisting of an Encoder (a contracting path to capture context) and a Decoder (an expanding path symmetric to the Encoder structure, that enables precise localization of structures) with skip connections between these. We refined the architecture using exponential linear units in the convolution layers instead of rectified linear units (ReLU) and a sigmoid function for the output. The network was implemented in Python (Version 3.5.2) using Tensorflow GPU (Version 1.2.0) and Keras (Verison 2.2.4). The training has been performed end-to-end, for 20–30 epochs using an Adam optimizer with a binary cross-entropy loss, a learning rate of 0.001 and a batch size of one.

To obtain the training data for the automatic segmentation, 1024 B-scans of four control and four tumor-injected zebrafish at different time points were manually segmented, based on the ground truth results obtained from histology. The segmentation was performed manually in 3D Slicer^60^. A 0.33 train/test split of the dataset was used resulting in 5 subjects (3 control and 2 tumor fish) for training and 3 subjects (1 control and 2 tumor fish) for testing. In total 640 slices (128 slices per subject) and corresponding target segmentations were used in the training. The evaluation was performed on 3 subjects (not included in the training) with a total number of 384 test images, resulting in a network segmentation accuracy of 99.66%.

The automatic generated segmentation data were used as binary masks and were applied to the scattering and polarization (birefringence and DOPU) images to analyze the distribution of these values quantitatively. For each volume 30 B-scans at the injection site were used to extract mean scattering, birefringence, and DOPU values.

All statistical evaluations were performed in MATLAB (MATLAB, R2021a, MathWorks). Box-whisker plots were created, showing the median values (red line), the 25–75% percentiles (box), the maximum and minimum values (horizontal bars) and outliers (red crosses) of the respective properties analyzed. Mann–Whitney U tests (rank sum test) were performed to test for the equality of the distributions of the data using a significance level of p< 0.05.

#### Birefringence abnormality analysis

Using the birefringence data an abnormality analysis was performed. Birefringence values smaller than 0.0006 were defined as abnormal. To define this value, a histogram analysis of selected tumor and control cases was performed. Utilizing the segmentation data, a percentage count of these abnormal low birefringence values in the tail muscle region was evaluated. Rank sum tests using a significance level of p< 0.05 were performed to identify statistically significant differences.

## Supplementary Information

Supplementary Information.

## Data Availability

The datasets generated during and/or analyzed during the current study are available from the corresponding author on reasonable request.

## References

[1] Wild C, Weiderpass E, Stewart B (2020). World Cancer Report: Cancer Research for Cancer Prevention. International Agency for Research on Cancer.

[2] Dai X (2015). Breast cancer intrinsic subtype classification, clinical use and future trends. Am. J. Cancer Res..

[3] Arnedos M (2015). Precision medicine for metastatic breast cancer-limitations and solutions. Nat. Rev. Clin. Oncol..

[4] Crimini E (2021). Precision medicine in breast cancer: From clinical trials to clinical practice. Cancer Treat. Rev..

[5] Costa B, Estrada MF, Mendes RV, Fior R (2020). Zebrafish avatars towards personalized medicine—a comparative review between avatar models. Cells.

[6] Baxendale S, Eeden FV, Wilkinson R (2017). The power of zebrafish in personalised medicine. Person. Med..

[7] Fazio M, Ablain J, Chuan Y, Langenau DM, Zon LI (2020). Zebrafish patient avatars in cancer biology and precision cancer therapy. Nat. Rev. Cancer.

[8] Goldsmith JR, Jobin C (2012). Think small: Zebrafish as a model system of human pathology. J. Biomed. Biotechnol..

[9] Kirchberger S, Sturtzel C, Pascoal S, Distel M (2017). Quo natas, Danio?—Recent progress in modeling cancer in zebrafish. Front. Oncol..

[10] Berghmans S (2005). Making waves in cancer research: New models in the zebrafish. Biotechniques.

[11] Gamble JT, Elson DJ, Greenwood JA, Tanguay RL, Kolluri SK (2021). The zebrafish xenograft models for investigating cancer and cancer therapeutics. Biology.

[12] Xiao J, Glasgow E, Agarwal S (2020). Zebrafish xenografts for drug discovery and personalized medicine. Trends Cancer.

[13] Yan C, Yang Q, Do D, Brunson DC, Langenau DM (2019). Adult immune compromised zebrafish for xenograft cell transplantation studies. EBioMedicine.

[14] Miller DR, Jarrett JW, Hassan AM, Dunn AK (2017). Deep tissue imaging with multiphoton fluorescence microscopy. Curr. Opin. Biomed. Eng..

[15] White RM (2008). Transparent adult zebrafish as a tool for in vivo transplantation analysis. Cell Stem Cell.

[16] Drexler W, Fujimoto J (2015). Optical Coherence Tomography: Technology and Applications.

[17] Makita S, Hong Y, Yamanari M, Yatagai T, Yasuno Y (2006). Optical coherence angiography. Opt. Express.

[18] De Boer JF, Hitzenberger CK, Yasuno Y (2017). Polarization sensitive optical coherence tomography—a review. Biomed. Opt. Express.

[19] Welzel J (2001). Optical coherence tomography in dermatology: A review. Skin Res. Technol. Rev. Article.

[20] Wang J, Xu Y, Boppart SA (2017). Review of optical coherence tomography in oncology. J. Biomed. Opt..

[21] Men J (2015). Optical coherence tomography for brain imaging and developmental biology. IEEE J. Sel. Top. Quantum Electron..

[22] Baumann B (2017). Polarization sensitive optical coherence tomography: A review of technology and applications. Appl. Sci..

[23] Kagemann L (2008). Repeated, noninvasive, high resolution spectral domain optical coherence tomography imaging of zebrafish embryos. Mol. Vis..

[24] Divakar Rao K, Upadhyaya P, Sharma M, Gupta P (2012). Noninvasive imaging of ethanol-induced developmental defects in zebrafish embryos using optical coherence tomography. Birth Defects Res. B.

[25] Chen Y, Trinh LA, Fingler J, Fraser SE (2016). Phase variance optical coherence microscopy for label-free imaging of the developing vasculature in zebrafish embryos. J. Biomed. Opt..

[26] Haindl R (2020). Functional optical coherence tomography and photoacoustic microscopy imaging for zebrafish larvae. Biomed. Opt. Express.

[27] Huckenpahler AL (2016). Imaging the adult zebrafish cone mosaic using optical coherence tomography. Visual Neurosci..

[28] Lapierre-Landry M (2018). Imaging melanin distribution in the zebrafish retina using photothermal optical coherence tomography. Trans. Vis. Sci. Technol..

[29] Bailey TJ, Davis DH, Vance JE, Hyde DR (2012). Spectral-domain optical coherence tomography as a noninvasive method to assess damaged and regenerating adult zebrafish retinas. Investig. Ophthalmol. Visual Sci..

[30] Rao KD, Alex A, Verma Y, Thampi S, Gupta PK (2009). Real-time in vivo imaging of adult zebrafish brain using optical coherence tomography. J. Biophoton..

[31] Lin Y-S, Chu C-C, Tsui P-H, Chang C-C (2013). Evaluation of zebrafish brain development using optical coherence tomography. J. Biophoton..

[32] Zhang J, Ge W, Yuan Z (2015). In vivo three-dimensional characterization of the adult zebrafish brain using a 1325 nm spectral-domain optical coherence tomography system with the 27 frame/s video rate. Biomed. Opt. Express.

[33] Yang D, Hu M, Zhang M, Liang Y (2020). High-resolution polarization-sensitive optical coherence tomography for zebrafish muscle imaging. Biomed. Opt. Express.

[34] Yang D, Yuan Z, Yang Z, Hu M, Liang Y (2021). High-resolution polarization-sensitive optical coherence tomography and optical coherence tomography angiography for zebrafish skin imaging. J. Innov. Opt. Health Sci..

[35] Lichtenegger A (2022). Multicontrast investigation of in vivo wildtype zebrafish in three development stages using polarization-sensitive optical coherence tomography. J. Biomed. Opt..

[36] Lichtenegger A (2022). Non-destructive characterization of adult zebrafish models using Jones matrix optical coherence tomography. Biomed. Opt. Express.

[37] Zudaire E, Gambardella L, Kurcz C, Vermeren S (2011). A computational tool for quantitative analysis of vascular networks. PLoS One.

[38] Pasquesi JJ (2006). In vivo detection of exercise-induced ultrastructural changes in genetically-altered murine skeletal muscle using polarization-sensitive optical coherence tomography. Opt. Express.

[39] Yang X (2013). Quantitative assessment of muscle damage in the mdx mouse model of Duchenne muscular dystrophy using polarization-sensitive optical coherence tomography. J. Appl. Physiol..

[40] Maierdiyali A, Wang L, Luo Y, Li Z (2020). Effect of tank size on zebrafish behavior and physiology. Animals.

[41] Owen JP, Kelsh RN (2021). A suitable anaesthetic protocol for metamorphic zebrafish. PLoS One.

[42] Wang J (2018). Complementary use of polarization-sensitive and standard OCT metrics for enhanced intraoperative differentiation of breast cancer. Biomed. Opt. Express.

[43] Stoletov K, Montel V, Lester RD, Gonias SL, Klemke R (2007). High-resolution imaging of the dynamic tumor cell-vascular interface in transparent zebrafish. Proc. Natl. Acad. Sci..

[44] Donnarumma D, Brodoline A, Alexandre D, Gross M (2018). Blood flow imaging in zebrafish by laser doppler digital holography. Microsc. Res. Tech..

[45] Mousavi SE, Patil JG (2020). Light-cardiogram, a simple technique for heart rate determination in adult zebrafish, Danio rerio. Comp. Biochem. Physiol. Part A Mol. Integrative Physiol..

[46] Huang W-C (2010). Combined use of MS-222 (tricaine) and isoflurane extends anesthesia time and minimizes cardiac rhythm side effects in adult zebrafish. Zebrafish.

[47] Jain RK (2003). Molecular regulation of vessel maturation. Nat. Med..

[48] Madu CO, Wang S, Madu CO, Lu Y (2020). Angiogenesis in breast cancer progression, diagnosis, and treatment. J. Cancer.

[49] Mendonça-Gomes JM (2021). Long-term dexamethasone treatment increases the engraftment efficiency of human breast cancer cells in adult zebrafish. Fish Shellf. Immunol. Rep..

[50] Ryu B, Je J-G, Jeon Y-J, Yang H-W (2021). Zebrafish model for studying dexamethasone-induced muscle atrophy and preventive effect of Maca (*Lepidium meyenii*). Cells.

[51] Kimmel CB, Ballard WW, Kimmel SR, Ullmann B, Schilling TF (1995). Stages of embryonic development of the zebrafish. Dev. Dyn..

[52] Li E, Makita S, Hong Y-J, Kasaragod D, Yasuno Y (2017). Three-dimensional multi-contrast imaging of in vivo human skin by Jones matrix optical coherence tomography. Biomed. Opt. Express.

[53] Schindelin J (2012). Biological imaging software tools. Nat. Methods.

[54] Makita S, Yamanari M, Yasuno Y (2010). Generalized Jones matrix optical coherence tomography: Performance and local birefringence imaging. Opt. Express.

[55] Kasaragod D (2014). Bayesian maximum likelihood estimator of phase retardation for quantitative polarization-sensitive optical coherence tomography. Opt. Express.

[56] Götzinger E (2008). Retinal pigment epithelium segmentation by polarization sensitive optical coherence tomography. Opt. Express.

[57] Makita S, Hong Y-J, Miura M, Yasuno Y (2014). Degree of polarization uniformity with high noise immunity using polarization-sensitive optical coherence tomography. Opt. Lett..

[58] Goel MK, Khanna P, Kishore J (2010). Understanding survival analysis: Kaplan–Meier estimate. Int. J. Ayurveda Res..

[59] Ronneberger, O., Fischer, P. & Brox, T. U-net: Convolutional networks for biomedical image segmentation. In *International Conference on Medical image computing and computer-assisted intervention*, 234–241 (Springer, 2015).

[60] Kikinis, R., Pieper, S. D. & Vosburgh, K. G. 3D Slicer: a platform for subject-specific image analysis, visualization, and clinical support. In *Intraoperative Imaging and Image-Guided Therapy*, 277–289 (Springer, 2014).

