# Supplementary material for: Longitudinal investigation of a xenograft tumor zebrafish model using polarization-sensitive optical coherence tomography
Source: Sci Rep. 2022 Sep 13;12:15381. doi: 10.1038/s41598-022-19483-z (PMC9470556; doi:10.1038/s41598-022-19483-z)
Supplement: Supplementary file 1 — Supplementary Information. [file 41598_2022_19483_MOESM1_ESM.pdf]

Supplementary material: Longitudinal investigation of a xenograft tumor zebrafish model using polarization-sensitive optical coherence tomography

Antonia Lichtenegger[1,2*], Junya Tamaoki[3], Roxane Licandro[4,5], Tomoko Mori[6], Pradipta Mukherjee[1], Lixuan Bian[3], Lisa Greutter[7], Shuichi Makita[1], Adelheid Wöhrer[7], Satoshi Matsusaka[6], Makoto Kobayashi[3], Bernhard Baumann[2], and Yoshiaki Yasuno[1]

[1]Computational Optics Group, University of Tsukuba, Japan
[2]Center for Medical Physics and Biomedical Engineering, Medical University of Vienna, Austria
[3]Department of Molecular and Developmental Biology, Faculty of Medicine, University of Tsukuba, Japan
[4]Computational Imaging Research Lab, Department of Biomedical Imaging and Image-guided Therapy, Medical University of Vienna, Vienna, Austria
[5]Laboratory for Computational Neuroimaging, Athinoula A. Martinos Center for Biomedical Imaging, Massachusetts General Hospital and Harvard Medical School, Charlestown, MA, USA
[6]Clinical Research and Regional Innovation, Faculty of Medicine University of Tsukuba, Japan
[7]Division of Neuropathology and Neurochemistry, Department of Neurology, Medical University of Vienna, Austria
[*]antonia.lichtenegger@cog-labs.org

Control zebrafish investigation

An additional cohort of control zebrafish (N = 8) with no injection were imaged with the JM-OCT prototype. The same measurement protocol as described in the Method section was utilized. AB (wild-type) zebrafish at the age of one-month-post-fertilization were investigated. Zebrafish were grown under standard conditions with a 14-hour day and 10-hour night cycle. The water, which was kept at 28.5 degrees Celsius, was changed every day. The zebrafish were immersed in Dexamethasone (10 μg/ml) for two days before being imaged with the JM-OCT prototype under anesthesia (Tricaine (0.16 mg/ml)). All animal experiments were performed in accordance with the animal study guidelines of the University of Tsukuba, Japan.

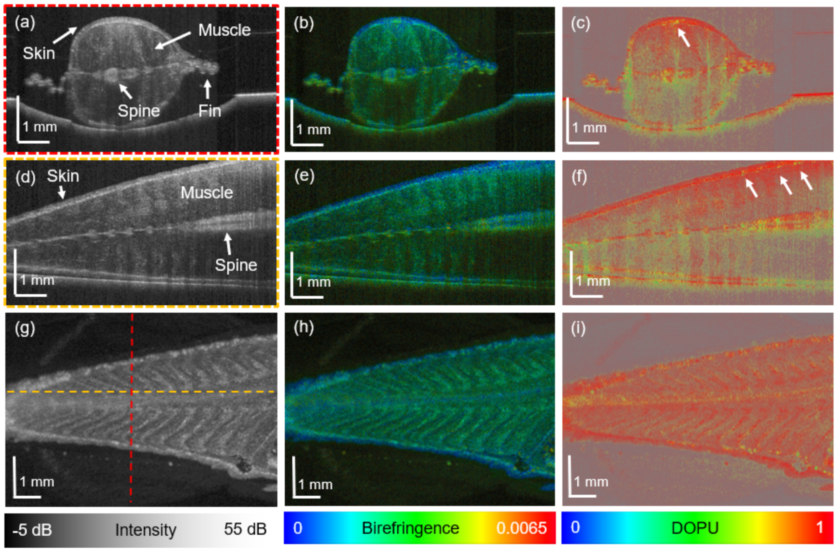

Figure S1. Imaging of control zebrafish at one-month post fertilization. (a) - (c) Transverse scatter-intensity, birefringence and DOPU B-scan images in the tail musculature region. (d) - (f) Corresponding sagittal sections. (g) - (i) Corresponding en-face images. The locations of the transverse and sagittal sections are indicated in (g) by red and orange dashed lines, respectively.

Representative JM-OCT results of one control fish are shown in Fig. S1. The intensity-based results are shown in Fig. S1 (a), (d) and (g), respectively. In the en-face image [Fig. S1 (g)] the locations of the transverse and sagittal sections, are indicated

by a red and an orange dashed line, respectively. The anatomical features of the fish such as the spine, the muscle region, the skin, and the fin can be identified.

The birefringence-based results are shown in Fig. S1 (b), (e) and (h), respectively. The musculature and the spine showed an increased birefringence signature. As described in our previous work the skin exhibits layers composed of low and high birefringence[1].

The DOPU-based images are presented in Fig. S1 (c), (f) and (i), respectively. The small regions of low DOPU values (indicated by white arrows) might correspond to more densely pigmented areas in the fish skin.

Muscle area evaluation

As described in the Result section in the manuscript a loss in muscle area was observed in control and tumor zebrafish over time in the second study. Representative Hematoxylin and Eosin stained histology micrographs of a control animal at 7 days post-injection (dpi) and 21 dpi are shown in Fig. S2 (a) and (b), respectively. Please note that multiple histology sections were obtained at various locations around the injection side in the fish tail, each showing similar appearances.

By utilizing the segmentation results obtained from the Zebrafish Segmentation Net, the change over time in average B-scan-wise (transverse section) muscle area (in the upper half) was examined for the JM-OCT data, see Fig, S2 (c). As described in the method section the 30 B-scans around the injection side were used for this evaluation. In the control animals, first a slight increase followed by a reduction in muscle area was observed. In the tumor-injected group, a decrease in muscle area was observed from 7 till 19 dpi. For both control and tumor injected zebrafish the muscle volume stayed rather constant low between 19 and 21 dpi.

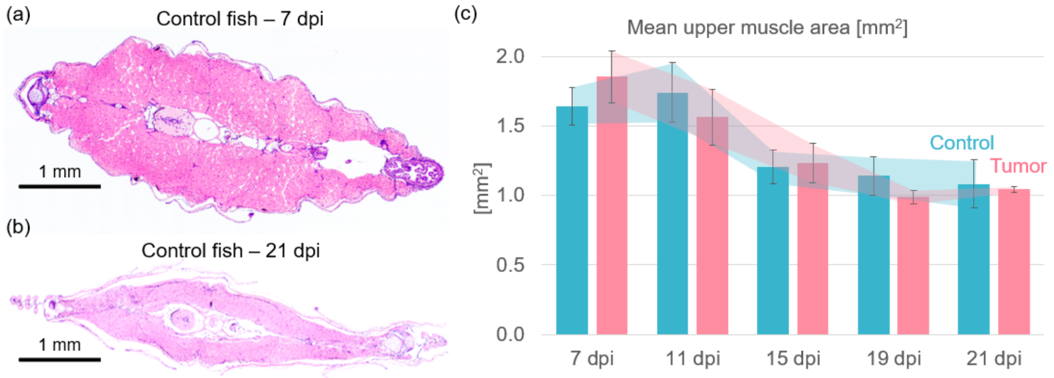

Figure S2. The muscle area changes over time. (a) - (b) Hematoxylin and Eosin-stained histology micrographs of a control fish at 7 and 9 days post injection (dpi). (c) Change over time in the average muscle area per transverse section, evaluated from the JM-OCT data. Standard deviations are indicated by vertical bars.

Immunohistochemistry staining results

To confirm the presents of the MCF-7 cells in the zebrafish tail musculature immunohistochemistry staining for breast cancer cell detection was performed. Figure S3 shows sagittal micrograph images obtained in the tail musculature of a representative control (a) - (b) and tumor-injected fish (c) - (d), respectively. In the control as well as in the tumor group unspecific staining (dark brown/black) was obtained which is due to formalin pigmentation, indicated by green arrows. In the tumor-injected fish the MCF-7 cells (brown) are highlighted by red arrows, see Fig. S3 (d).

Longitudinal imaging of xenograft zebrafish

By keeping the zebrafish in six well plates individual animals could be examined in a longitudinal fashion over 21-days. Figure S4 presents scatter and birefringence-based transverse cross-section images of a representative control and tumor-injected animal. The tumor locations are indicated by red arrows.

References

1. Lichtenegger, A. *et al.* Multicontrast investigation of in vivo wildtype zebrafish in three development stages using polarization-sensitive optical coherence tomography. *J. Biomed. Opt.* **27**, 016001 (2022).

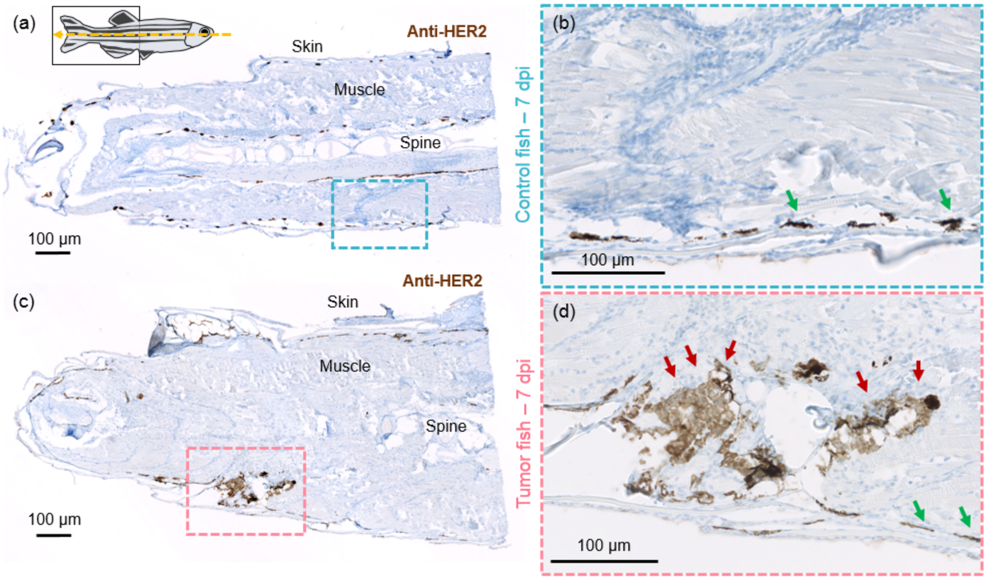

Figure S3. The immunohistochemistry stained micrographs. (a) Sagittal section in the tail region of a control fish 7 days post injection (dpi). (b) Zoom-in image into the muscle area. The unspecific staining (dark brown/black spots), indicated by green arrows, is due to formalin pigmentation and can be found in control and tumor-injected zebrafish. (c) Sagittal micrograph of a tumor-injected animal 7 dpi with a zoom-in image (d) into the tumor region. The MCF-7 human breast cancer cells were specifically stained (brown color) and are indicated by red arrows.

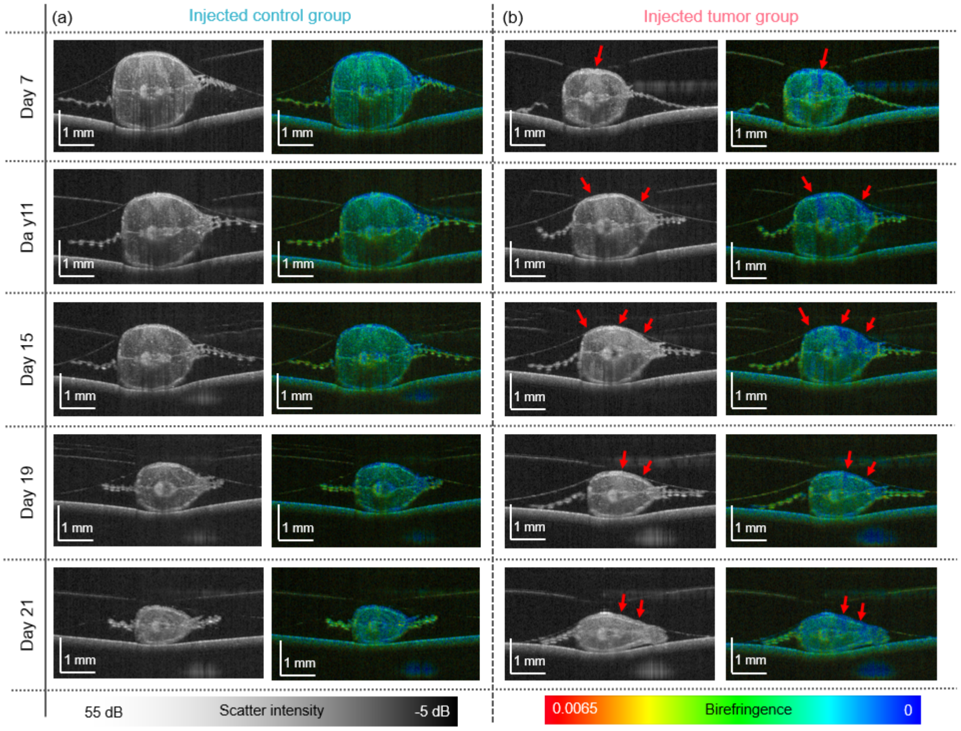

Figure S4. Longitudinal imaging of a control (a) and a tumor injected zebrafish (b). Cross-sectional intensity and birefringence-based JM-OCT results.
